# Self-Efficacy, Optimism, and Academic Performance as Psychoeducational Variables: Mediation Approach in Students

**DOI:** 10.3390/children9030420

**Published:** 2022-03-15

**Authors:** Pablo Usán, Carlos Salavera, Alberto Quílez-Robres

**Affiliations:** 1 Faculty of Human Sciences and Education, University of Zaragoza, 22003 Huesca, Spain; 2 Faculty of Education, University of Zaragoza, 50009 Zaragoza, Spain

**Keywords:** self-efficacy, optimism, academic performance, students, adolescents

## Abstract

**Background:** During the various stages of education, adolescents undergo emotional and motivational experiences that can play key roles in their development. This study aims to analyse the relationship among academic self-efficacy, optimism, and academic performance. **Methods:** This study comprised 1852 adolescent (male, *N* = 956, 51.61% and female, *N* = 896, 48.38%) aged 12–19 years (M = 14.77; SD = 1.80) from twelve secondary schools in Spain. The instruments used for the evaluation were the *Academic Self-Efficacy Scale* (ASES) and the *Life Orientation Test*—*Revised* (LOT-R); the students’ average marks were used to measure their academic performance. **Results:** The results of the study revealed significant correlations among self-efficacy, optimism and academic performance. **Conclusions:** These results emphasise the importance of academic self-efficacy as a mediating variable between the other two variables as well as its central role in the promotion of adaptive behaviours in the classroom, leading to adequate personal development, helping to prevent early school dropout and contributing to a more satisfactory academic experience.

## 1. Introduction

During students’ school years, they face numerous personal and contextual situations that can have a significant impact on their development, especially during adolescence, a key period for their academic and personal growth in the life cycle, when adult personality is forged [1].

Some students will pass this stage of life without difficulties at the academic and personal levels, but others, conditioned by various variables, may stagnate at some point and be affected by some psychological variables that in turn affect their academic performance [2].

For this reason, research on certain psychological variables can cause a positive impact in the school context and help in understanding the cognitive and motivational processes that can lead to personal and academic improvements [3].

Self-efficacy plays an important role in the learning process and in the way students face their school tasks [4]. In academic contexts, self-efficacy is a self-regulatory mechanism that affects the academic behaviour of students in that it determines the student’s perception of their own competence towards a given task and their ability to adapt to and cope with future academic demands [5].

As such, students with higher levels of self-efficacy perceive homework to be a challenge to be faced with confidence in their own ability and to practice their skills responsibly and efficiently [6]. Empirical research has shown that academic self-efficacy can be used to predict students’ participation and commitment towards school tasks [7]; perseverance and motivation [8]; academic performance [9]; and more broadly, greater levels of academic satisfaction and happiness [10]. Conversely, low levels of self-efficacy have been related to low commitment and poor academic performance [11] and have even lead to psychological conditions such as anxiety and stress [12,13].

Another important variable for self-perception in a variety of contexts is optimism, defined as a more or less stable set of positive expectations concerning future experiences [14].

Optimism is a personal pre-disposition that mediates between external conditions and the way we interpret them, affecting decision-making processes. While optimistic individuals tend to respond positively to adverse events, hence overcoming them, less optimistic individuals are less capable of responding to negative, critical, and even traumatic experiences [15].

The existing literature on optimism in academic contexts established that this factor can be used to predict other psychological variables. Optimistic students who use adaptive coping strategies [16] and present higher levels of personal and academic self-efficacy [17] are more likely to meet their personal and academic targets [18], are less vulnerable [19], present higher levels of self-concept and self-esteem, and are more assertive [20,21,22].

In short, optimism plays a crucial role in the way individuals confront everyday experiences, especially during adolescence, during which their adult personality is formed [14].

Finally, academic performance is understood as the quantitative/qualitative assessment of academic achievements during the learning process [23]. The scientific literature has been based mainly on two measures that assess and determine the academic performance of students: from a quantitative point of view, obtaining school grades and, from a more qualitative point on view, focused on their personal variables and context [24].

On the one hand, average for school grades has always served as a representative evaluation of students’ academic performance [25]. On the other hand, certain authors defend other types of evaluations as being better representatives of academic performance such as the number of repeated school years and even the time dedicated to student assimilation [26,27].

Academic performance in adolescents is a broad construct that has been approached from some perspectives and theoretical references. Gónzalez [28] emphasises the intrapersonal factors that determine the personality of adolescent students; Fierro, Almagro, and Sáenz-López [29] focus on all of those socio-emotional variables, especially the motivational processes that direct the behaviour of students. Pulido and Herrera [30] attached importance to the value of the influence of sociodemographic variables in the context closest to the student body.

For all of these reasons, following the study by Méndez [31], studies that advocate the interactive processes of students are necessary to learn first-hand about other variables that directly influence the academic performance of students not only to improve their grades but also to work with all of the underlying variables that affect personal development in students [32].

In this way, there are not many manuscripts that relate study variables to academic performance or other variables that play important roles in the study of factors that determine academic performance. Therefore, the objective of this research was to analyse the relationship among the variables self-efficacy, optimism, and academic performance in adolescent students.

The two main hypotheses of this investigation were as follows:

(a) Self-efficacy is linked to optimism and academic performance, stimulating adaptive behaviours;
(b) The relationship between optimism and students’ academic performance will be mediated by self-efficacy.

## 2. Materials and Methods

### 2.1. Sample

The study population comprised 1852 students (male, *N* = 956, 51.61% and female, *N* = 896, 48.38%) in 12 schools, with ages ranging 12–19 years (M = 14.77; SD = 1.80). The inclusion criteria were the ability to read and communicate, as a necessary condition to understand the questionnaires. Incomplete questionnaires (29) and students with cognitive disorders, which hampered a complete understanding of the questionnaires, were excluded. The different schools in the sample were chosen by simple random sampling through the completion of a questionnaire; 98.89% of questionnaires were returned and counted.

First, with the objective to evaluate the self-efficacy of students, the *Academic Self-Efficacy Scale* (ASES) was used, validated by García, Inglés, Torregrosa, Ruiz, Díaz, Pérez, and Martínez (2010) for adolescent students [33]. The scale comprises 10 individual items to evaluate self-efficacy in an academic context (for example, “I am convinced that I can carry out outstanding exams”). The responses to the questionnaire ranged from 1 to 5 points on a Likert-type scale, where 1 point means “Strongly disagree” (1) and 5 points means “Strongly agree” (5). The value of Cronbach’s alpha was 0.91, demonstrating high reliability in school environments, resulting in a Cronbach’s alpha in our study of 0.89.

Concerning optimism, Scheier, Carver, and Bridges’s (1994) [34] *Life Orientation Test*—*Revised* (LOT-R) was translated and validated into Spanish for adolescents by Ferrando, Chico, and Tous (2002) [35]. The scale includes six items, three of which are positive statements (for example, “I am always optimistic about my future”) and the other three being negative statements (for example, “I hardly ever expect things to go my way”). The answers to the questions were determined on a scale from 1 to 5 points on a Likert-type scale, where 1 point means “Strongly disagree” (1) and 5 points means “Strongly agree” (5). The value of Cronbach’s alpha was 0.78, demonstrating high reliability in academic and school environments, resulting in a Cronbach’s alpha in our study of 0.79.

Finally, the variable academic performance was evaluated on the basis of the average marks in the first trimester, ranging from 0 to 10 (minimum/maximum). This variable is widely used and regarded as an effective predictor of students’ academic performance [25,36,37]. For our study, this variable yielded a Cronbach-α of 0.86.

### 2.2. Protocol

The questionnaires were handed out to the students in their classrooms. All students from each school receiving the questionnaire on the same day provided signed informed consent from the parents/guardians in advance, coordinated with the school’s management. At all times, parents and students were informed of the objectives of the investigation and that their participation was voluntary, which is in line with the ethical directives set out in the Declaration of Helsinki (2000) [38]. The protocol was endorsed by the Opiics Research Group (46_20R, 2020–2022) (Psychology and Sociology Deparment of he University of Zaragoza, Zaragoza, 5009). Questionnaires were anonymous and confidential, and students could opt out at any point in the process.

### 2.3. Data Analysis

First, to establish the sociodemographic data of the students, descriptive statistics of the variables sex, age, academic year, type of study, and number of repetitions for the academic year and the variables self-efficacy, optimism, and academic performance were analyzed. After performing the descriptive analysis, bivariate correlations were made between the three study variables using IBM SPSS v26.0. Subsequently, a cluster analysis was executed to compare the sample in three significant groups, with each other using K-means in a cluster. Finally, a bootstrapping mediation analysis (10,000 runs) was performed in order to give the proposed model adequacy and confidence using a confidence level of 5% and *p* ≤ 0.05 at the significance.

## 3. Results

### 3.1. Demographic Variables of the Study

The study comprised 1852 adolescent (male, *N* = 956, 51.61% and female, *N* = 896, 48.38%) aged 12–19 years (M = 14.77; SD = 1.80), as shown in Table 1.

### 3.2. Descriptive Variables of the Study

The values for the variables self-efficacy, optimism, and academic performance are highly variable, as illustrated in Table 2. 

Optimism was shown to be of little statistical significance and slightly higher in males (Cohen’s d = 0.285). Males scored slightly higher in the variable self-efficacy, while females scored slightly higher in academic performance.

### 3.3. Correlational Analysis of the Study

As seen in Table 3, we can observe some correlations in all variables, which are related in different ways. Self-efficacy is positively correlated with optimism (*r* = 0.375) and academic performance (*r* = 0.370), while optimism showed a much weaker correlation with academic performance (*r* = 0.106) than self-efficacy (*r* = 0.375)

### 3.4. Cluster Analysis of Statistically Significant Groups of the Students

Three significant groups were made through a K-means cluster analysis to divide the students into three significant groups among them.

Group nº 1 (*N* = 596, 32.18%) was characterised by low scores in self-efficacy, optimism, and academic performance; Group º2 (*N* = 616, 33.26%) presented variable scores, with near average scores in self-efficacy, high scores in optimism, and poor academic performance; finally, Group nº 3 (*N* = 640, 34.55%) was characterised by high scores in self-efficacy, optimism, and academic performance, as illustrated in Table 4.

### 3.5. Mediation of the Effects of Self-Efficacy between Optimism and Academic Performance of Students

To establish whether the relationship between optimism and academic performance is mediated by self-efficacy, we used MACRO by Hayes (2018) [39] in SPSS Process 3.0 (v 26.0), continuing the methodology proposed by Tal-Or, Cohen, Tsarfati, and Gunther (2010) [40].

**Figure 1:**
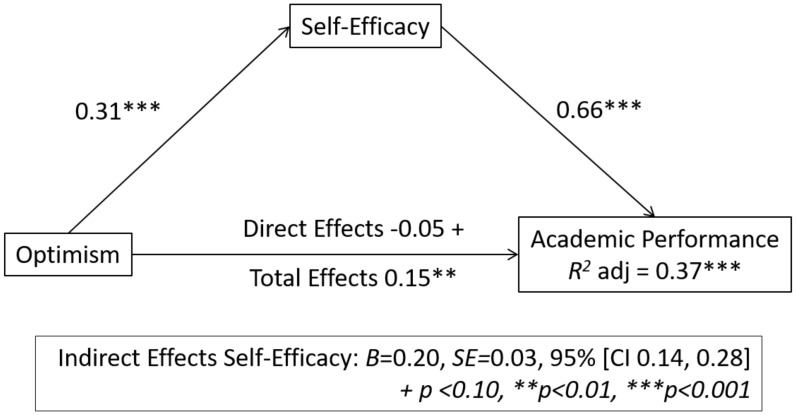
Values of the mediating role of self-efficacy in the variables of the study.

In Figure 1, we can see that self-efficacy plays a significant mediating effect on the relationship between optimism and academic achievement. Thus, the results indicate a mediating effect of optimism (VI) on self-efficacy of 0.31 m and a mediating effect of self-efficacy on academic performance (VD) of 0.66; in both cases, *p* > 0.001, which therefore, is statistically significant. Zero was not included in the bootstrap interval, B = 0.20, SE = 0.03, and 95% [CI 0.14, 0.28], so it can be stated that mean self-efficacy has a positive relationship with optimism and academic performance.

These findings establish that optimism by itself does not have a direct significant effect on academic performance (−0.05, *p* < 0.10) but that its combination with self-efficacy does, yielding a result of 0.15, *p* < 0.001 (direct effect + indirect effect), with the proportion of variance being explained by the model R2 = 0.37 ***.

These results suggest the role that self-efficacy plays in its relationship with optimism and academic performance, and the practical implications that it may entail.

## 4. Discussion

The results of our research aimed to analyse the intrinsic relationship among self-efficacy, optimism, and academic performance on a sample of school-age adolescents.

The first hypothesis raised was confirmed based on our findings. Namely, the results show that self-efficacy is positively correlated with optimism and academic performance.

Our finding is in line with other studies in the scientific literature. Alejos (2018) [41] established a relationship between academic self-efficacy and optimism as key factors in student happiness; De Besa, Gil, and García (2019) [42] showed that various individual psycho-social features determine the level of optimism, such as self-efficacy and learning strategies; Rand (2018) [43] showed the close relationship between optimism and self-efficacy in adolescent students in a study that dealt with self-confidence and hopes for the future; Fínez and Morán [44] drew a link between intrapersonal self-evaluation factors, such as self-concept, self-esteem, and self-efficacy, and resilience in adolescent students; finally, Liu et al. (2018) [45] pointed out that self-efficacy and optimism can be used to predict student subjective wellbeing.

On the other hand, the relation between self-efficacy and academic performance has been paid greater attention in a scholarly context. Some studies establish a direct relationship between these variables; Avalos, Oropeza, Ramírez, and Palos (2018) [46], and Galleguillos and Olmedo (2017) [47] found statistically significant correlations between self-efficacy and academic performance; Castro (2020) [48] drew a similar link in both primary and secondary school contexts. 

Other studies have examined the relationship between self-efficacy and academic performance in broader studies that take into consideration more variables, such as study strategies, coping styles, resilience and self-esteem, the development of creativity, and commitment and motivation towards school tasks [9,28,49,50].

The second hypothesis that we made in the investigation was also confirmed; that is, self-efficacy remained as a mediating variable between optimism and academic performance. In this way, we can affirm that the academic self-efficacy of the adolescent students in the study influences the relationship between the other two variables. However, these results must be carefully commented on since, although they present bidirectional correlations with each other, optimism is a poor predictor of academic performance; that is, the effect of the former on the latter is not statistically significant.

The current scientific literature is divided about this issue. Some studies find that optimism can predict academic performance: for example, Pulido and Herrera (2018) [51] in a sample of secondary school students and Kolovelonies and Goudas (2018) in primary school students, among others [52,53,54]. Other studies, however, argue against the predictive value of optimism over academic performance [17,55,56], in agreement with our own results.

The results suggest that self-efficacy plays a mediating role between the other two variables, which only emphasises the importance of this variable for adolescent students, especially by mediating between optimism and academic performance. This has relevant practical implications.

There are some investigation that examine these variables from different points of view, but no previous investigation have directly addressed the mediating role of self-efficacy in the relationship with optimism and academic performance. Alhadabi and Karpinski (2020) [57] established the mediating role of self-efficacy between more intrinsically oriented academic motivations and school performance; Usher, Li, Butz, and Rojas (2019) [58] reached similar conclusions with a sample of students in different educational tiers; Avalos, Oropeza, Ramírez, and Palos (2018) [46] established the mediating role of self-efficacy in the relationship with academic skills and achievements; Cerezo et al. (2019) [59] established a direct correlation between training in self-regulated learning strategies and an increased understanding of said strategies, a relationship in which self-efficacy plays a mediating role. Alipio (2020) [60] alluded to the influence of self-efficacy on value expectancy beliefs about students’ academic performance. Udavar, Fiori, and Bausseron (2020) [61] found that the relationship between achievement and emotional intelligence was supported by the modulation of the self-efficacy of adolescents.

## 5. Conclusions

For these reasons, the important role played by the constructs used in our research is highlighted, which together with the personal and contextual circumstances of students, can directly affect school performance [62].

The limitations of this study are chiefly related to its lateral nature. Data collection was a one-off event, and as a result, the data have no temporal depth, while scores can easily change significantly from year to year and even within the same school year. In a similar fashion, the schools were randomly selected in terms of type of school, students and teachers, socioeconomic conditions, and social/cultural settings.

Future studies should examine the role that self-efficacy plays for students as well as its influence on other psychological variables. That students feel competent in their school tasks is a prerequisite for other adaptive behaviours and an adequate personal and academic development. It is also necessary to undertake longitudinal studies that allow for an examination of the evolution of these constructs over a longer time span, although the methodological challenges that these studies pose must be recognised. In addition, it would be interesting to take into consideration other academic tiers, such as primary school (6–11 years) and university (18 years and over). It would also be of interest to take into consideration other sociodemographic variables, such as gender, age, and type of school (public, private, and rural environments). Similarly, programmes directed by psychology and educational professionals can also help to improve students’ overall experience, decreasing the risk of early school dropout.

## Figures and Tables

**Table 1:** Sociodemographic features of the sample.

		*N*	%
Gender (Male-Female)	Male	956	51.58
	Female	896	48.41
Years (12–17)	12	208	11.23
	13	272	14.68
	14	279	15.06
	15	421	21.38
	16	396	18.92
	17	172	9.28
	18	83	4.48
	19	21	1.14
Academic year * (1ºESO–2ª BACH)	1º ESO *	263	14.20
	2º ESO	326	17.60
	3º ESO	286	15.44
	4º ESO	494	26.67
	1º BACH	373	20.14
	2º BACH	110	5.93
Repeated course (Yes–No)	Yes	420	22.67
	No	1432	77.32
Type of school (Public-Private)	Public	1204	65.01
	Private	648	34.98

* Refers to Spanish stages (ESO: 11/12–15/16 years old)/(BACH 16/17–18–19 years old).

**Table 2:** Descriptive values of the variables.

	Total		Male		Female		
	x	sd	x	sd	x	sd	Cohen’s d
Self-Efficacy	3.24	0.68	3.28	0.68	3.20	0.68	0.117
Optimism	3.56	0.81	3.68	0.77	3.45	0.84	0.285
Academic Performance	2.92	1.17	2.85	1.16	2.98	1.19	−0.110

**Table 3:** Values of the correlational analysis.

	1	2	3
Self-Efficacy	1		
Optimism	0.375 **	1	
Academic Performance	0.370 **	0.106 *	1
Mean (X)	3.24	3.56	2.98
SD	0.68	0.81	1.17
Cronbach’s alpha	0.89	0.79	0.86

** Correlation significant at the 0.01 level. * Correlation significant at the 0.05 level.

**Table 4:** Values of the cluster analysis.

	Total Sample		Group nº 1 (*N* = 596, 32.18%)		Group nº 2 (*N* = 616, 33.26%)		Group nº 3 (*N* = 640, 34.55%)	
	x	sd	x	sd	x	sd	x	sd
Self-Efficacy	3.24	0.68	2.59	0.52	3.29	0.46	3.79	0.44
Optimism	3.56	0.81	2.75	0.63	3.92	0.55	3.97	0.60
Academic Performance	2.92	1.17	2.59	1.12	2.12	0.90	3.99	0.45

## Data Availability

The data presented in this study are available on request from the corresponding autor.
